# Supplementary material for: Risk of dispersion or aerosol generation and infection transmission with nasopharyngeal and oropharyngeal swabs for detection of COVID-19: a systematic review
Source: BMJ Open. 2021 Mar 17;11(3):e040616. doi: 10.1136/bmjopen-2020-040616 (PMC7977073; doi:10.1136/bmjopen-2020-040616)
Supplement: Supplementary data [file bmjopen-2020-040616supp001.pdf]

Appendix A: Search strategy for OVID Medline and EMBASE.

MEDLINE
Database: OVID Medline Epub Ahead of Print, In-Process & Other Non-Indexed Citations, Ovid MEDLINE(R) Daily and Ovid MEDLINE(R) 1946 to Present
Search Strategy:
----
1 exp Respiratory Tract Infections/ (352559)
2 (respiratory adj3 infect*).mp. [mp=title, abstract, original title, name of substance word, subject heading word, floating sub-heading word, keyword heading word, organism supplementary concept word, protocol supplementary concept word, rare disease supplementary concept word, unique identifier, synonyms] (75025)
3 (influenza* or flu).tw. (121359)
4 (h1n1 or h5n1 or h3n2).tw. (25097)
5 exp Coronavirus/ (11467)
6 exp Coronavirus Infections/ (9776)
7 (coronavir* or coronovir* or SARS or MERS or MERS-COV or SARS-COV or SARS-COV-2 or COV or NCOV or 2019nCOV or 2019-nCOV or COVID-19).mp. [mp=title, abstract, original title, name of substance word, subject heading word, floating sub-heading word, keyword heading word, organism supplementary concept word, protocol supplementary concept word, rare disease supplementary concept word, unique identifier, synonyms] (24352)
8 or/1-7 (469619)
9 ((throat or pharynx or pharyng* or oral or oropharyng* or nasal or nares or nasopharyng*) adj3 (swab* or detect* or test or sample* or specimen* or diagnos* or assay)).mp. (49581)
10 8 and 9 (8147)
11 exp Disease Transmission, Infectious/ (67129)
12 (contamina* or dispers* or droplet* or partic* or aerosol* or environment* or transmiss* or transmit or safety* or commun* or contagi*).mp. (5570978)
13 11 or 12 (5584372)
14 10 and 13 (2488)

EMBASE
Database: Embase <1974 to 2020 March 24>
Search Strategy:
----
1 exp respiratory tract infection/ (385263)
2 (respiratory adj3 infect*).mp. (127592)
3 (influenza* or flu).tw. (139944)
4 (h1n1 or h5n1 or h3n2).tw. (30836)
5 exp coronavirinae/ (11209)
6 exp Coronavirus infection/ (11152)
7 (coronavir* or coronovir* or SARS or MERS or MERS-COV or SARS-COV or SARS-COV-2 or COV or NCOV or 2019nCOV or 2019-nCOV or COVID-19).mp. (28959)
8 or/1-7 (491703)
9 ((throat or pharynx or pharyng* or oral or oropharyng* or nasal or nares or nasopharyng*) adj3 (swab* or detect* or test or sample* or specimen* or diagnos* or assay)).mp. (80034)

10 8 and 9 (9252)
11 disease transmission/ (96856)
12 (contamina* or dispers* or droplet* or partic* or aerosol* or environment* or transmiss* or transmit or safety* or commun* or contagi*).mp. (7223561)
13 11 or 12 (7223561)
14 10 and 13 (3189)
