# Supplementary material for: Risk of dispersion or aerosol generation and infection transmission with nasopharyngeal and oropharyngeal swabs for detection of COVID-19: a systematic review
Source: BMJ Open. 2021 Mar 17;11(3):e040616. doi: 10.1136/bmjopen-2020-040616 (PMC7977073; doi:10.1136/bmjopen-2020-040616)
Supplement: Author's manuscript [file bmjopen-2020-040616.draft_revisions.pdf]

BMJ Open is committed to open peer review. As part of this commitment we make the peer review history of every article we publish publicly available.

When an article is published we post the peer reviewers' comments and the authors' responses online. We also post the versions of the paper that were used during peer review. These are the versions that the peer review comments apply to.

The versions of the paper that follow are the versions that were submitted during the peer review process. They are not the versions of record or the final published versions. They should not be cited or distributed as the published version of this manuscript.

BMJ Open is an open access journal and the full, final, typeset and author-corrected version of record of the manuscript is available on our site with no access controls, subscription charges or pay-per-view fees (http://bmjopen.bmj.com).

If you have any questions on BMJ Open's open peer review process please email info.bmjopen@bmj.com

BMJ Open

Risk of dispersion or aerosol generation and infection transmission with nasopharyngeal and oropharyngeal swabs for detection of COVID-19: a systematic review

Journal:	*BMJ Open*
Manuscript ID	bmjopen-2020-040616
Article Type:	Original research
Date Submitted by the Author:	18-May-2020
Complete List of Authors:	Agarwal, Arnav; University of Toronto, Department of Medicine<br>Fernando, Shannon; University of Ottawa, Department of Emergency Medicine; University of Ottawa, Division of Critical Care, Department of Medicine<br>Honarmand, Kimia; Western University, 4. Division of Critical Care, Department of Medicine, Schulich School of Medicine and Dentistry<br>Bakaa, Layla; McMaster University, Faculty of Science<br>Brar, Sonia; University at Buffalo - The State University of New York, School of Medicine and Biomedical Sciences<br>Granton, David; McMaster University, Michael G. DeGroote School of Medicine<br>Chaudhuri, Dipayan; McMaster University, Department of Medicine<br>Chetan, Devin; University of Toronto, Department of Pediatrics; Hospital for Sick Children, Division of Cardiology, Labatt Family Heart Centre<br>Hu, Malini; McMaster University, Michael G. DeGroote School of Medicine<br>Basmaji, John; Western University, Division of Critical Care, Department of Medicine<br>Muttalib, Fiona; Hospital for Sick Children, Center for Global Child Health<br>Rochwerg, Bram; McMaster University, Michael G. DeGroote School of Medicine; McMaster University, Department of Health Research Methods, Evidence and Impact<br>Adhikari, Neill; Sunnybrook Health Sciences Centre, Department of Critical Care Medicine; University of Toronto, Interdepartmental Division of Critical Care Medicine<br>Lamontagne, Francois; Universite de Sherbrooke; Centre de recherche du CHU de Sherbrooke<br>Hui, David; Chinese University of Hong Kong Faculty of Medicine, Department of Medicine and Therapeutics; Chinese University of Hong Kong Faculty of Medicine, Stanley Ho Center for Emerging Infectious Diseases<br>Gomersall, Charles; Chinese University of Hong Kong Faculty of Medicine, Department of Anaesthesia and Intensive Care<br>Mubareka, Samira; Sunnybrook Health Sciences Centre, Department of Laboratory Medicine and Pathobiology; University of Toronto, Division of Infectious Diseases<br>Diaz, Janet; World Health Organization; California Pacific Medical Center<br>Burns, Karen; University of Toronto, Interdepartmental Division of Critical Care Medicine; Unity Health Toronto, Department of Critical Care Medicine

	Couban, Rachel; McMaster University, Michael G. DeGroote Institute for Pain Research and Care; McMaster University, Department of Health Research Methods, Evidence and Impact<br>Vandvik, Per; MAGIC Evidence Ecosystem Foundation

Keywords:	Diagnostic microbiology < INFECTIOUS DISEASES, Infection control < INFECTIOUS DISEASES, Public health < INFECTIOUS DISEASES

SCHOLARONE™
Manuscripts

I, the Submitting Author has the right to grant and does grant on behalf of all authors of the Work (as defined in the below author licence), an exclusive licence and/or a non-exclusive licence for contributions from authors who are: i) UK Crown employees; ii) where BMJ has agreed a CC-BY licence shall apply, and/or iii) in accordance with the terms applicable for US Federal Government officers or employees acting as part of their official duties; on a worldwide, perpetual, irrevocable, royalty-free basis to BMJ Publishing Group Ltd ("BMJ") its licensees and where the relevant Journal is co-owned by BMJ to the co-owners of the Journal, to publish the Work in this journal and any other BMJ products and to exploit all rights, as set out in our licence.

The Submitting Author accepts and understands that any supply made under these terms is made by BMJ to the Submitting Author unless you are acting as an employee on behalf of your employer or a postgraduate student of an affiliated institution which is paying any applicable article publishing charge ("APC") for Open Access articles. Where the Submitting Author wishes to make the Work available on an Open Access basis (and intends to pay the relevant APC), the terms of reuse of such Open Access shall be governed by a Creative Commons licence – details of these licences and which Creative Commons licence will apply to this Work are set out in our licence referred to above.

Other than as permitted in any relevant BMJ Author's Self Archiving Policies, I confirm this Work has not been accepted for publication elsewhere, is not being considered for publication elsewhere and does not duplicate material already published. I confirm all authors consent to publication of this Work and authorise the granting of this licence.

For peer review only

Risk of dispersion or aerosol generation and infection transmission with nasopharyngeal and oropharyngeal swabs for detection of COVID-19: a systematic review.

Arnav Agarwal,[1] Shannon M. Fernando,[2,3] Kimia Honarmand,[4] Layla Bakaa,[5] Sonia Brar,[6] David Granton,[7] Dipayan Chaudhuri,[8] Devin Chetan,[9,10] Malini Hu,[7] John Basmaji,[4] Fiona Muttalib,[11] Bram Rochwerg,[7,12] Neill K. Adhikari,[13,14] Francois Lamontagne,[15,16] Srinivas Murthy,[17] David S.C. Hui,[18,19] Charles Gomersall,[20] Samira Mubareka,[21,22] Janet V. Diaz,[23,24] Karen E. A. Burns,[14,,25,26] Rachel Couban,[12,27] Per O. Vandvik.[28]

1. Department of Medicine, University of Toronto, Toronto, Canada.
2. Division of Critical Care, Department of Medicine, University of Ottawa, Ottawa, Canada
3. Department of Emergency Medicine, University of Ottawa, Ottawa, Canada.
4. Division of Critical Care, Department of Medicine, Schulich School of Medicine and Dentistry, Western University, London, Canada.
5. Faculty of Science, McMaster University, Hamilton, Canada.
6. School of Medicine and Biomedical Sciences, University of Buffalo, Buffalo, NY, United States.
7. Michael G. DeGroote School of Medicine, McMaster University, Hamilton, Canada.
8. Department of Medicine, McMaster University, Hamilton, Canada.
9. Department of Pediatrics, University of Toronto, Toronto, Canada.
10. Division of Cardiology, Labatt Family Heart Centre, The Hospital for Sick Children, Toronto, Canada.
11. Center for Global Child Health, Hospital for Sick Children, Toronto, Canada.
12. Department of Health Research Methods, Evidence and Impact, McMaster University, Hamilton, Canada.
13. Department of Critical Care Medicine, Sunnybrook Health Sciences Centre, Toronto, Canada
14. Interdepartmental Division of Critical Care Medicine, University of Toronto, Toronto, Canada
15. Professor, Université de Sherbrooke, Sherbrooke, Canada
16. 2) Clinician Scientist, Centre de recherche du CHU de Sherbrooke, Sherbrooke, Canada
17. BC Children's Hospital, University of British Columbia, Vancouver, Canada
18. Dept of Medicine and Therapeutics, The Chinese University of Hong Kong, Shatin, Hong Kong SAR, China.
19. Stanley Ho Center for Emerging Infectious Diseases, The Chinese University of Hong Kong, Shatin, Hong Kong SAR, China.
20. Department of Anaesthesia and Intensive Care, The Chinese University of Hong Kong, Hong Kong
21. Division of Infectious Diseases, University of Toronto, Toronto, Canada.
22. Department of Laboratory Medicine and Pathobiology, University of Toronto, Toronto, Canada.
23. Pacific Medical Center, San Francisco, CA, USA
24. World Health Organization, Geneva, Switzerland
25. Unity Health Toronto – St. Michael's Hospital, Toronto, Canada
26. Li Ka Shing Knowledge Institute, Toronto, Canada
27. Michael G. DeGroote Institute for Pain Research and Care, McMaster University, Hamilton, Canada.
28. MAGIC Evidence Ecosystem Foundation, Oslo, Norway

Corresponding author:

Per Olav Vandvik, MD, Ph.D, professor
MAGIC Evidence Ecosystem Foundation
Phone: +47 975 123 91 e-mail: per.vandvik@gmail.com

Word Count: 1,560.

Keywords: nasopharyngeal, oropharyngeal, swabs, aerosol generation, airborne transmission, SARS-CoV-2, COVID-19.

For peer review only

Abstract:

Objectives: Severe acute respiratory syndrome coronavirus 2 (SARS-CoV-2)-related disease, referred to as COVID-19, has emerged as a global pandemic since December 2019. Although transmission is primarily through respiratory droplets and close contact, airborne transmission may be possible in the setting of aerosol generating procedures and treatments. Whether nasopharyngeal and oropharyngeal swabs for COVID-19 generate aerosols remains unclear. This rapid systematic review commissioned by the World Health Organization aimed to determine the risk of aerosol generation and associated transmission with nasopharyngeal and oropharyngeal swabs for detection of COVID-19.

Design: Systematic review.

Data Sources: We searched Ovid MEDLINE and EMBASE from inception until March 25 2020. We also searched the China National Knowledge Infrastructure, Chinese Medical Journal Network, medRxiv and ClinicalTrials.gov as of March 29 2020.

Eligibility Criteria: All comparative and non-comparative studies that evaluated dispersion or aerosolization of viable airborne organisms or transmission of infection associated with nasopharyngeal or oropharyngeal swab testing.

Results: Of 6,640 citations, we retrieved 6 full-text articles. After careful full-text review, no citations were deemed eligible for inclusion.

Conclusions: Aerosol generating procedures may expose healthcare workers and other contacts to pathogens and increase risk of transmission. There is no existing data regarding aerosol generation and risk of transmission with nasopharyngeal and oropharyngeal swabs for the detection of COVID-19. Field experiments to quantify this risk are warranted. Current international guidelines recommend strict droplet and contact precautions during testing.

For peer review only

Vigilance in adhering to current standards for infection prevention and control measures is suggested.

For peer review only

Article summary:

- Aerosolization and associated transmission of COVID-19 and other respiratory infections may be possible in the setting of aerosol generating procedures.
- This risk of airborne transmission to uninfected individuals and healthcare workers must be balanced with severe resource constraints in personal protective equipment.
- The risk of aerosol generation associated with nasopharyngeal and oropharyngeal swabs for COVID-19 and other respiratory infections remains unclear, precluding reliable evidence-informed guidance.

Strengths and limitations of this study:

- A comprehensive literature search incorporating English and Chinese-language peer reviewed literature, grey literature and pre-print databases was conducted.
- There was no direct or indirect evidence identified.

For peer review only

Background:

In late December 2019, a novel coronavirus, subsequently referred to by the World Health Organization (WHO) as *severe acute respiratory syndrome coronavirus 2* (SARS-CoV-2), was identified as the cause of atypical pneumonia cases detected in Wuhan, China.[1] Since then, SARS-CoV-2 related-disease (named COVID-19), has emerged as a global pandemic.[2] As of May 17, SARS-CoV-2 has spread to over 200 countries, infecting 4.7 million individuals, and causing over 313,000 deaths internationally.[3]

COVID-19 is primarily transmitted through mucosal contact with respiratory droplets (5 to 10 μm in diameter) either through close contact (within 1 meter) with an individual who has respiratory symptoms, or through indirect contact with contaminated surfaces.[1,4,5] Airborne transmission may be possible in specific circumstances and settings in which potential aerosol generating procedures or treatments are performed, such as tracheal intubation and bronchoscopy.[5]

On March 29th, 2020, the WHO issued a scientific brief recommending droplet and contact precautions for those people caring for COVID-19 patients and airborne precautions for settings in which aerosol generating procedures and treatments are performed, according to risk assessment.[5] However, crucial knowledge gaps persist regarding which interventions and procedures are associated with clinically significant aerosolization.

Testing for COVID-19 is commonly conducted using nasopharyngeal and oropharyngeal swabs of the posterior wall of the pharynx. The risk of aerosol generation associated with obtaining nasopharyngeal or oropharyngeal swabs in patients infected with COVID-19 remains unclear. The potential to induce coughing may compound this associated risk, the extent to which is unknown.[6]

Given thousands of nasopharyngeal and oropharyngeal swabs are conducted daily internationally, the risk of airborne transmission to otherwise uninfected patients and healthcare

For peer review only

workers must be balanced with severe resource constraints in personal protective equipment. This dictates an urgent need for an updated evidence synthesis and guidance regarding the risk of aerosolization associated with swab testing. We therefore conducted a rapid systematic review of the literature to determine the risk of aerosol generation and associated transmission associated with nasopharyngeal and oropharyngeal swabs for detection of COVID-19.

For peer review only

Methods:

Prior to beginning, WHO personnel reviewed and approved internal protocols for this systematic review. Given time constraints of the commissioned review (7 days to completion), the protocol was not registered or published.

Literature search: We conducted a comprehensive search of Ovid MEDLINE and Embase with the assistance of a health information specialist (RC) from inception to March 25, using a combination of subject headings and keywords related to: respiratory tract infections including COVID-19 and other coronaviruses; swabs related to the oral and nasal cavities, nasopharynx and oropharynx; and aerosol generation and infection transmission. We did not limit the search to COVID-19 or coronavirus infection (see Appendix A for the search strategy).

We also applied the same search strategy to the China National Knowledge Infrastructure (CNKI) and Chinese Medical Journal Network (CMJN) (YC). We did not apply any language or quality restrictions. Finally, we searched medRxiv and ClinicalTrials.gov for articles related to COVID-19 or SARS-CoV-2 for pre-prints or grey literature addressing the research question.

Selection criteria: We included all comparative and non-comparative studies that evaluated dispersion or aerosolization of viable airborne organisms or transmission of infection associated with nasopharyngeal or oropharyngeal swab testing. We included studies that evaluated outcomes including detection of viable airborne organisms through microbiological sample analysis or documented transmission of infection associated with swab testing. We initially planned to include studies of hospitalized adult and pediatric patients with microbiologically confirmed COVID-19 in one or more fluid samples, of which at least one was a nasopharyngeal or oropharyngeal swab. In the absence of direct evidence meeting the eligibility criteria above, we broadened our inclusion

For peer review only

criteria to include all study designs and populations evaluating aerosol generation or dispersion associated with swab testing. This included studies of hospitalized and non-hospitalized patients with or without microbiologically confirmed COVID-19, simulation studies without human participants, and those describing dispersion of non-infectious air particles or liquid droplets.

Study selection: Paired reviewers (SMF, KH, LB, SB) independently screened citations and conducted full-text review of all potentially eligible studies. Reviewers screened the reference lists of review articles to identify additional studies meeting eligibility criteria, and screened pre-prints and grey literature from medRxiv and ClinicalTrials.gov. Disagreements were resolved by a third reviewer (AA) at all stages. Paired reviewers (XX, NY, XL) screened citations identified from the CNKI and CMJN and resolved disagreements by discussion.

Data extraction and quality assessment: Paired reviewers planned to abstract data using a standardized form. We planned to assess risk of bias (RoB) using the modified Cochrane risk of bias tool for randomized controlled trials,[7] the ROBINS-I tool for observational studies,[8] and content expert assessments for experimental or simulation-based studies. We planned to assess the overall certainty of the evidence based on RoB, imprecision, indirectness, inconsistency and publication bias, informed by GRADE guidance.[9,10]

Data analysis: If insufficient data were available for pooled analyses, we planned to summarize results narratively.

For peer review only

Results:

Of the 6640 citations identified in our search, 4762 were screened for eligibility after duplicates were removed. Of these, 6 were considered potentially eligible requiring full-text review. After careful full-text review, we did not identify any citations evaluating aerosol generation related to nasopharyngeal or oropharyngeal swab testing despite broadening eligibility criteria (Figure 1).

For peer review only

Discussion

Our systematic review on aerosol generation and associated risk of infection transmission with nasopharyngeal and oropharyngeal swabs in the detection of COVID-19 yielded no relevant studies. Broadening of eligibility criteria to allow for all study designs, including non-comparative and experimental designs and all populations regardless of confirmation of COVID-19 infection, also did not yield any relevant citations.

Airborne transmission is different from droplet transmission, as it refers to the presence of microbes within droplet nuclei. These are generally considered to be particles <5μm in diameter, which can remain in the air for periods of time, and can subsequently be transmitted to others over distances greater than 1 meter.[5] Reduction of respiratory particles to <5 μm involves dehydration of larger droplets and consequent dehydration of any organisms within the droplet. If airborne particles are inhaled, they may rehydrate in the upper airway, increase in size and deposit in the airway. Thus, airborne transmission of viable organisms requires the organism to survive a process of desiccation and aerosolization in sufficient quantity to incite infection. This property is specific to the organism.

Aerosol generating procedures may expose healthcare workers and other contacts to pathogens, and the potential for transmission of respiratory infections. The risk of aerosolization and transmission with swab testing remains unclear, particularly in the context of COVID-19. The most important theoretical risk associated with testing is induction of coughing with possible droplet release and aerosolization. A previous study evaluating voluntary cough-generated aerosolization of influenza virus using a bioaerosol cyclone sampler found that 81% of participants produced detectable viral RNA, with 23% of the RNA contained in particles 1 to 4 micrometres in size and 42% contained in particles less than 1 micrometre.[11] Observational data from Bischoff

and colleagues showed that patients with confirmed influenza may expose health care workers within 1.83 m to infectious doses of influenza-inoculated droplets.[12] This evidence has guided the WHO to recommend a droplet safety distance of two metres for respiratory illnesses with possible droplet transmission by contact, and appropriate precautions for healthcare workers in close contact with individuals with potential or confirmed infection. COVID-19 has been shown to maintain stability for up to 3 hours in aerosols[13], although this data is based on experimentally induced aerosol-generating procedure with unknown relevance to the clinical setting.

Taken together, recommendations regarding infection prevention and control measures during nasopharyngeal or oropharyngeal swab testing for COVID-19 should be carefully considered in light of the unknown risk of airborne transmission of infection to healthcare workers and other patients. Field experiments with nasopharyngeal and oropharyngeal swab testing in confirmed COVID-19 patients are required to adequately assess this risk using viral samplers, reverse transcriptase polymerase chain reaction testing, and viral cultures. While we cannot estimate the risk of airborne transmission, certain factors may abrogate it: adequate room ventilation, limiting exposure to the patient, high filtration fit-tested respirators (e.g. N95, FFP2) and low viral shedding.[14] However, the risks ought to be weighed against limited resource availability and the need for judicious use of personal protective equipment. One may also consider the yield of various fluid samples, and balance their associated risks of aerosolization with this consideration. The emergence of a diagnostic test with no potential for aerosol generation emerge (e.g. serology) may diminish the enthusiasm for nasopharyngeal and oropharyngeal swabs.

Strengths of this systematic review include a comprehensive literature search incorporating English and Chinese studies, as well as grey literature and pre-prints and consultation with experts in the field. Limitations include a lack of evidence informing the risk of aerosolization with

For peer review only

nasopharyngeal and oropharyngeal swab testing, including indirect evidence. This highlights an important gap in the existing literature and precludes development of evidence-informed guidance beyond recommendations based on expert opinion.

For peer review only

Conclusion

There is no evidence regarding the risk of aerosol generation and airborne transmission associated with nasopharyngeal and oropharyngeal swab testing in the context of COVID-19 or related infectious respiratory illnesses. Current international guidelines recommend strict droplet and contact precautions during testing. Given a theoretical risk of cough-related aerosolization during testing, field experiments to quantify the risk of aerosol generation and transmission associated with swab testing are warranted. In the interim, appropriate vigilance in adhering to available standards for infection prevention and control measures is suggested.

For peer review only

Funding: This project was supported by the World Health Organization as part of a rapid review effort related to COVID-19, through the MAGIC Evidence Ecosystem Foundation (www.magicproject.org).

Acknowledgements: We would like to thank Yaolong Chen, Xuan Yu, Nan Yang and Xufei Luo for their assistance with systematic searching and screening of evidence from the China National Knowledge Infrastructure database for additional literature related to our systematic review focused on risk of aerosol generation with HFNC.

Author Contributions: POV conceived the study. AA and POV organized the study teams and process. AA and RC designed the search strategy. SMF, KH, LB, and SB screened studies for eligibility, and assessed study RoB and certainty of the body of evidence. AA addressed discrepancies in screening. AA wrote the first draft of the manuscript and conducted data analysis. AA, BR, NA, FM, SM, DSH, CG, SM, JVD, KB, JB, FM, DG, DC1, DC2, MH, and POV critically revised the manuscript. AA and POV are the guarantors. All co-authors were involved in final editing of the manuscript, and approved the final draft. The corresponding author attests that all listed authors meet authorship criteria.

Patient and public involvement: No patients were involved in this study, given the severe time constraints of 7 days to completion for this WHO-commissioned rapid review.

Data sharing statement: No additional data available.

Ethics approval statement: Not applicable.

Transparency: The manuscript's guarantors (POV and AA) affirm that the manuscript is an honest, accurate, and transparent account of the recommendation being reported; that no important aspects of the recommendation have been omitted; and that any discrepancies from the recommendation as planned (and, if relevant, registered) have been explained.

For peer review only

For peer review only

References

1. Zhu N, Zhang D, Wang W, et al. A novel coronavirus from patients with pneumonia in China, 2019. N Engl J Med. 2020;382:727-33.
2. World Health Organization. Rolling updates on coronavirus disease (COVID-19) [Internet]. 2020 Mar 23 [cited 2020 Mar 24]. Available from: https://www.who.int/emergencies/diseases/novel-coronavirus-2019/events-as-they-happen
3. John Hopkins University of Medicine Coronavirus Resource Centre. Coronavirus COVID-19 global cases by the Center for Systems Science and Engineering (CSSE) at John Hopkins University. 2020 May 17 [cited 2020 May 17]. Available from: https://coronavirus.jhu.edu/map.html
4. Wang W, Xu Y, Gao R, et al. Detection of SARS-CoV-2 in different types of clinical specimens. JAMA. 2020 Mar 11 [Epub ahead of print].
5. World Health Organization. Modes of transmission of virus causing COVID-19: implications for IPC precaution recommendations [Internet]. 2020 Mar 29 [cited 2020 Mar 29]. Available from: https://www.who.int/news-room/commentaries/detail/modes-of-transmission-of-virus-causing-covid-19-implications-for-ipc-precaution-recommendations
6. World Health Organization. Q&A on infection prevention and control for health care workers caring for patients with suspected or confirmed 2019-nCoV [Internet]. 2020 Mar 01 [cited 2020 Mar 31]. Available from: https://www.who.int/news-room/q-a-detail/q-a-on-infection-prevention-and-control-for-health-care-workers-caring-for-patients-with-suspected-or-confirmed-2019-ncov.

For peer review only

7. Higgins JPT, Altman DG, Gotzsche PC, et al. The Cochrane Collaboration's tool for assessing risk of bias in randomised trials. BMJ. 2011;343:d5928.

8. Sterne JA, Hernán MA, Reeves BC, et al. ROBINS-I: a tool for assessing risk of bias in non-randomised studies of interventions. BMJ. 2016;355:i4919.

9. Guyatt GH, Oxman AD, Vist GE, et al. GRADE: an emerging consensus on rating quality of evidence and strength of recommendations. BMJ (Clinical Research ed). 2008;336(7650):924-6.

10. Morgan RL, Thayer KA, Bero L, et al. GRADE: Assessing the quality of evidence in environmental and occupational health. Environ Int. 2016;92-93:611-6.

11. Lindsley WG, Blachere FM, Thewlis RE, et al. Measurements of airborne influenza virus in aerosol particles from human coughs. PLoS One. 2010;5(11):e15100.

12. Bischoff WE, Swett K, Leng I, Peters TR. Exposure to influenza virus aerosols during routine patient care. J Infect Dis. 2013;207(7):1037-46.

13. van Doremalen N, Bushmaker T, Morris DH, et al. Aerosol and surface stability of SARS-CoV-2 as compared with SARS-CoV-1. N Engl J Med. 2020 [Epub ahead of print].

14. Fennelly KP, Nardell EA. The relative efficacy of respirators and room ventilation in preventing occupational tuberculosis. Infect Cont Hosp Ep. 1998;19:754-9.

For peer review only

Figure 1: PRISMA flow diagram for review on aerosol generation associated with nasopharyngeal and oropharyngeal swabs.

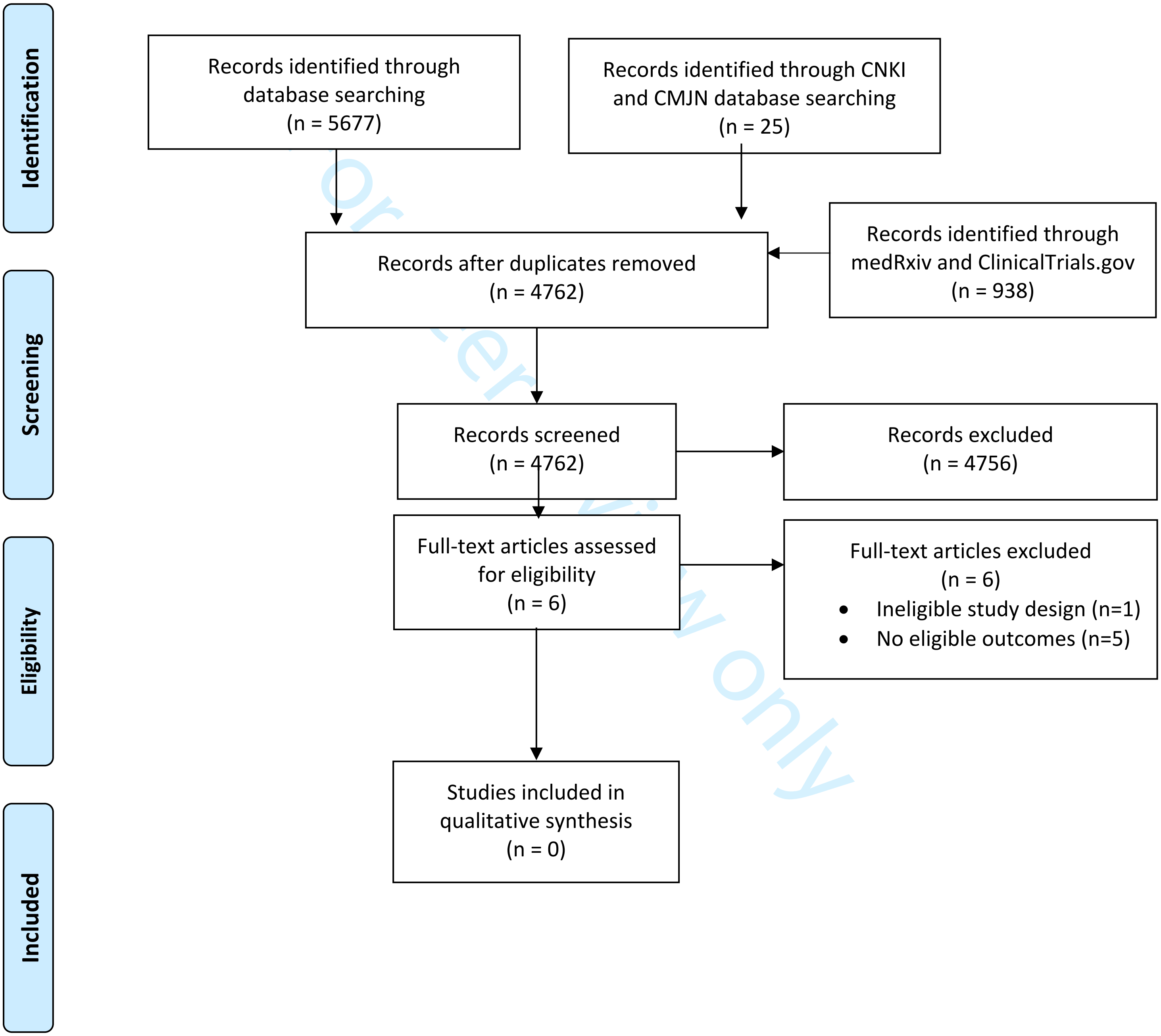

For peer review only

Appendix A: Search strategy for OVID Medline and EMBASE.

MEDLINE
Database: OVID Medline Epub Ahead of Print, In-Process & Other Non-Indexed Citations, Ovid MEDLINE(R) Daily and Ovid MEDLINE(R) 1946 to Present
Search Strategy:

----

1 exp Respiratory Tract Infections/ (352559)
2 (respiratory adj3 infect*).mp. [mp=title, abstract, original title, name of substance word, subject heading word, floating sub-heading word, keyword heading word, organism supplementary concept word, protocol supplementary concept word, rare disease supplementary concept word, unique identifier, synonyms] (75025)
3 (influenza* or flu).tw. (121359)
4 (h1n1 or h5n1 or h3n2).tw. (25097)
5 exp Coronavirus/ (11467)
6 exp Coronavirus Infections/ (9776)
7 (coronavir* or coronovir* or SARS or MERS or MERS-COV or SARS-COV or SARS-COV-2 or COV or NCOV or 2019nCOV or 2019-nCOV or COVID-19).mp. [mp=title, abstract, original title, name of substance word, subject heading word, floating sub-heading word, keyword heading word, organism supplementary concept word, protocol supplementary concept word, rare disease supplementary concept word, unique identifier, synonyms] (24352)
8 or/1-7 (469619)
9 ((throat or pharynx or pharyng* or oral or oropharyng* or nasal or nares or nasopharyng*) adj3 (swab* or detect* or test or sample* or specimen* or diagnos* or assay)).mp. (49581)
10 8 and 9 (8147)
11 exp Disease Transmission, Infectious/ (67129)
12 (contamina* or dispers* or droplet* or partic* or aerosol* or environment* or transmiss* or transmit or safety* or commun* or contagi*).mp. (5570978)
13 11 or 12 (5584372)
14 10 and 13 (2488)

EMBASE
Database: Embase <1974 to 2020 March 24>
Search Strategy:

----

1 exp respiratory tract infection/ (385263)
2 (respiratory adj3 infect*).mp. (127592)
3 (influenza* or flu).tw. (139944)
4 (h1n1 or h5n1 or h3n2).tw. (30836)
5 exp coronavirinae/ (11209)
6 exp Coronavirus infection/ (11152)
7 (coronavir* or coronovir* or SARS or MERS or MERS-COV or SARS-COV or SARS-COV-2 or COV or NCOV or 2019nCOV or 2019-nCOV or COVID-19).mp. (28959)
8 or/1-7 (491703)
9 ((throat or pharynx or pharyng* or oral or oropharyng* or nasal or nares or nasopharyng*) adj3 (swab* or detect* or test or sample* or specimen* or diagnos* or assay)).mp. (80034)

For peer review only

10 8 and 9 (9252)
11 disease transmission/ (96856)
12 (contamina* or dispers* or droplet* or partic* or aerosol* or environment* or transmiss* or transmit or safety* or commun* or contagi*).mp. (7223561)
13 11 or 12 (7223561)
14 10 and 13 (3189)

For peer review only

PRISMA 2009 Checklist

Section/topic	#	Checklist item	Reported on page #
**TITLE**			
Title	1	Identify the report as a systematic review, meta-analysis, or both.	1
**ABSTRACT**			
Structured summary	2	Provide a structured summary including, as applicable: background; objectives; data sources; study eligibility criteria, participants, and interventions; study appraisal and synthesis methods; results; limitations; conclusions and implications of key findings; systematic review registration number.	2-4
**INTRODUCTION**			
Rationale	3	Describe the rationale for the review in the context of what is already known.	5-6
Objectives	4	Provide an explicit statement of questions being addressed with reference to participants, interventions, comparisons, outcomes, and study design (PICOS).	5-6
**METHODS**			
Protocol and registration	5	Indicate if a review protocol exists, if and where it can be accessed (e.g., Web address), and, if available, provide registration information including registration number.	7
Eligibility criteria	6	Specify study characteristics (e.g., PICOS, length of follow-up) and report characteristics (e.g., years considered, language, publication status) used as criteria for eligibility, giving rationale.	7
Information sources	7	Describe all information sources (e.g., databases with dates of coverage, contact with study authors to identify additional studies) in the search and date last searched.	7
Search	8	Present full electronic search strategy for at least one database, including any limits used, such that it could be repeated.	7
Study selection	9	State the process for selecting studies (i.e., screening, eligibility, included in systematic review, and, if applicable, included in the meta-analysis).	7
Data collection process	10	Describe method of data extraction from reports (e.g., piloted forms, independently, in duplicate) and any processes for obtaining and confirming data from investigators.	8
Data items	11	List and define all variables for which data were sought (e.g., PICOS, funding sources) and any assumptions and simplifications made.	8
Risk of bias in individual studies	12	Describe methods used for assessing risk of bias of individual studies (including specification of whether this was done at the study or outcome level), and how this information is to be used in any data synthesis.	8
Summary measures	13	State the principal summary measures (e.g., risk ratio, difference in means).	8
Synthesis of results	14	Describe the methods of handling data and combining results of studies, if done, including measures of consistency (e.g., I^2) for each meta-analysis.	8

For peer review only - http://bmjopen.bmj.com/site/about/guidelines.xhtml

PRISMA 2009 Checklist

Section/topic	#	Checklist item	Reported on page #
Risk of bias across studies	15	Specify any assessment of risk of bias that may affect the cumulative evidence (e.g., publication bias, selective reporting within studies).	8
Additional analyses	16	Describe methods of additional analyses (e.g., sensitivity or subgroup analyses, meta-regression), if done, indicating which were pre-specified.	N/A
**RESULTS**			
Study selection	17	Give numbers of studies screened, assessed for eligibility, and included in the review, with reasons for exclusions at each stage, ideally with a flow diagram.	9
Study characteristics	18	For each study, present characteristics for which data were extracted (e.g., study size, PICOS, follow-up period) and provide the citations.	N/A
Risk of bias within studies	19	Present data on risk of bias of each study and, if available, any outcome level assessment (see item 12).	N/A
Results of individual studies	20	For all outcomes considered (benefits or harms), present, for each study: (a) simple summary data for each intervention group (b) effect estimates and confidence intervals, ideally with a forest plot.	N/A
Synthesis of results	21	Present results of each meta-analysis done, including confidence intervals and measures of consistency.	N/A
Risk of bias across studies	22	Present results of any assessment of risk of bias across studies (see Item 15).	N/A
Additional analysis	23	Give results of additional analyses, if done (e.g., sensitivity or subgroup analyses, meta-regression [see Item 16]).	N/A
**DISCUSSION**			
Summary of evidence	24	Summarize the main findings including the strength of evidence for each main outcome; consider their relevance to key groups (e.g., healthcare providers, users, and policy makers).	9
Limitations	25	Discuss limitations at study and outcome level (e.g., risk of bias), and at review-level (e.g., incomplete retrieval of identified research, reporting bias).	9
Conclusions	26	Provide a general interpretation of the results in the context of other evidence, and implications for future research.	9-11
**FUNDING**			
Funding	27	Describe sources of funding for the systematic review and other support (e.g., supply of data); role of funders for the systematic review.	12

From: Moher D, Liberati A, Tetzlaff J, Altman DG, The PRISMA Group (2009). Preferred Reporting Items for Systematic Reviews and Meta-Analyses: The PRISMA Statement. PLoS Med 6(7): e1000097. doi:10.1371/journal.pmed1000097

For more information, visit: **www.prisma-statement.org**.

For peer review only - http://bmjopen.bmj.com/site/about/guidelines.xhtml

BMJ Open

Risk of dispersion or aerosol generation and infection transmission with nasopharyngeal and oropharyngeal swabs for detection of COVID-19: a systematic review

Journal:	*BMJ Open*
Manuscript ID	bmjopen-2020-040616.R1
Article Type:	Original research
Date Submitted by the Author:	25-Jan-2021

Complete List of Authors:	Agarwal, Arnav; University of Toronto, Department of Medicine<br>Fernando, Shannon; University of Ottawa, Department of Emergency Medicine; University of Ottawa, Division of Critical Care, Department of Medicine<br>Honarmand, Kimia; Western University, 4. Division of Critical Care, Department of Medicine, Schulich School of Medicine and Dentistry<br>Bakaa, Layla; McMaster University, Faculty of Science<br>Brar, Sonia; University at Buffalo - The State University of New York, School of Medicine and Biomedical Sciences<br>Granton, David; McMaster University, Michael G. DeGroote School of Medicine<br>Chaudhuri, Dipayan; McMaster University, Department of Medicine<br>Chetan, Devin; University of Toronto, Department of Pediatrics; Hospital for Sick Children, Division of Cardiology, Labatt Family Heart Centre<br>Hu, Malini; McMaster University, Michael G. DeGroote School of Medicine<br>Basmaji, John; Western University, Division of Critical Care, Department of Medicine<br>Muttalib, Fiona; Hospital for Sick Children, Center for Global Child Health<br>Rochwerg, Bram; McMaster University, Michael G. DeGroote School of Medicine; McMaster University, Department of Health Research Methods, Evidence and Impact<br>Adhikari, Neill; Sunnybrook Health Sciences Centre, Department of Critical Care Medicine; University of Toronto, Interdepartmental Division of Critical Care Medicine<br>Lamontagne, Francois; Universite de Sherbrooke, Department of Medicine and Centre de Recherche du CHUS<br>Murthy, Srinivas; University of British Columbia, Departments of Pediatrics and Critical Care Medicine<br>Hui, David; Chinese University of Hong Kong Faculty of Medicine, Department of Medicine and Therapeutics; Chinese University of Hong Kong Faculty of Medicine, Stanley Ho Center for Emerging Infectious Diseases<br>Gomersall, Charles; Chinese University of Hong Kong Faculty of Medicine, Department of Anaesthesia and Intensive Care<br>Mubareka, Samira; Sunnybrook Health Sciences Centre, Department of Laboratory Medicine and Pathobiology; University of Toronto, Division of Infectious Diseases<br>Diaz, Janet; World Health Organization; California Pacific Medical Center<br>Burns, Karen; University of Toronto, Interdepartmental Division of

	Critical Care Medicine; Unity Health Toronto, Department of Critical Care Medicine<br>Couban, Rachel; McMaster University, Michael G. DeGroote Institute for Pain Research and Care; McMaster University, Department of Health Research Methods, Evidence and Impact<br>Vandvik, Per; Norwegian Institute of Public Health; University of Oslo, Institute of Health and Society
**Primary Subject Heading**:	Public health
Secondary Subject Heading:	Diagnostics, Infectious diseases, Public health
Keywords:	Diagnostic microbiology < INFECTIOUS DISEASES, Infection control < INFECTIOUS DISEASES, Public health < INFECTIOUS DISEASES

SCHOLARONE™
Manuscripts

I, the Submitting Author has the right to grant and does grant on behalf of all authors of the Work (as defined in the below author licence), an exclusive licence and/or a non-exclusive licence for contributions from authors who are: i) UK Crown employees; ii) where BMJ has agreed a CC-BY licence shall apply, and/or iii) in accordance with the terms applicable for US Federal Government officers or employees acting as part of their official duties; on a worldwide, perpetual, irrevocable, royalty-free basis to BMJ Publishing Group Ltd ("BMJ") its licensees and where the relevant Journal is co-owned by BMJ to the co-owners of the Journal, to publish the Work in this journal and any other BMJ products and to exploit all rights, as set out in our licence.

The Submitting Author accepts and understands that any supply made under these terms is made by BMJ to the Submitting Author unless you are acting as an employee on behalf of your employer or a postgraduate student of an affiliated institution which is paying any applicable article publishing charge ("APC") for Open Access articles. Where the Submitting Author wishes to make the Work available on an Open Access basis (and intends to pay the relevant APC), the terms of reuse of such Open Access shall be governed by a Creative Commons licence – details of these licences and which Creative Commons licence will apply to this Work are set out in our licence referred to above.

Other than as permitted in any relevant BMJ Author's Self Archiving Policies, I confirm this Work has not been accepted for publication elsewhere, is not being considered for publication elsewhere and does not duplicate material already published. I confirm all authors consent to publication of this Work and authorise the granting of this licence.

For peer review only

Risk of dispersion or aerosol generation and infection transmission with nasopharyngeal and oropharyngeal swabs for detection of COVID-19: a systematic review.

Arnav Agarwal,[1] Shannon M. Fernando,[2,3] Kimia Honarmand,[4] Layla Bakaa,[5] Sonia Brar,[6] David Granton,[7] Dipayan Chaudhuri,[8] Devin Chetan,[9,10] Malini Hu,[7] John Basmaji,[4] Fiona Muttalib,[11] Bram Rochwerg,[7,12] Neill K. Adhikari,[13,14] Francois Lamontagne,[15,16] Srinivas Murthy,[17] David S.C. Hui,[18,19] Charles D. Gomersall,[20] Samira Mubareka,[21,22] Janet V. Diaz,[23,24] Karen E. A. Burns,[14,,25,26] Rachel Couban,[12,27] Per O. Vandvik.[28]

1. Department of Medicine, University of Toronto, Toronto, Canada.
2. Division of Critical Care, Department of Medicine, University of Ottawa, Ottawa, Canada
3. Department of Emergency Medicine, University of Ottawa, Ottawa, Canada.
4. Division of Critical Care, Department of Medicine, Western University, London, Canada.
5. Faculty of Science, McMaster University, Hamilton, Canada.
6. School of Medicine and Biomedical Sciences, University of Buffalo, Buffalo, NY, United States.
7. Michael G. DeGroote School of Medicine, McMaster University, Hamilton, Canada.
8. Department of Medicine, McMaster University, Hamilton, Canada.
9. Department of Pediatrics, University of Toronto, Toronto, Canada.
10. Division of Cardiology, Labatt Family Heart Centre, The Hospital for Sick Children, Toronto, Canada.
11. Center for Global Child Health, Hospital for Sick Children, Toronto, Canada.
12. Department of Health Research Methods, Evidence and Impact, McMaster University, Hamilton, Canada.
13. Department of Critical Care Medicine, Sunnybrook Health Sciences Centre, Toronto, Canada
14. Interdepartmental Division of Critical Care Medicine, University of Toronto, Toronto, Canada
15. Université de Sherbrooke, Sherbrooke, Canada
16. Centre de recherche du CHU de Sherbrooke, Sherbrooke, Canada
17. BC Children's Hospital, University of British Columbia, Vancouver, Canada
18. Department of Medicine and Therapeutics, The Chinese University of Hong Kong, Shatin, Hong Kong SAR, China.
19. Stanley Ho Center for Emerging Infectious Diseases, The Chinese University of Hong Kong, Shatin, Hong Kong SAR, China.
20. Department of Anaesthesia and Intensive Care, The Chinese University of Hong Kong, Hong Kong
21. Division of Infectious Diseases, University of Toronto, Toronto, Canada.
22. Department of Laboratory Medicine and Pathobiology, University of Toronto, Toronto, Canada.
23. Pacific Medical Center, San Francisco, CA, USA
24. World Health Organization, Geneva, Switzerland
25. Unity Health Toronto – St. Michael's Hospital, Toronto, Canada
26. Li Ka Shing Knowledge Institute, Toronto, Canada
27. Michael G. DeGroote Institute for Pain Research and Care, McMaster University, Hamilton, Canada.
28. MAGIC Evidence Ecosystem Foundation, Oslo, Norway

Corresponding author:

Per Olav Vandvik, MD, Ph.D, professor
Institute of Health and Society, University of
MAGIC Evidence Ecosystem Foundation
Phone: +47 975 123 91 e-mail: per.vandvik@gmail.com

Word Count: 1,943.

Keywords: nasopharyngeal, oropharyngeal, swabs, aerosol generation, airborne transmission, SARS-CoV-2, COVID-19.

For peer review only

Abstract:

Objectives: Severe acute respiratory syndrome coronavirus 2 (SARS-CoV-2)-related disease, referred to as COVID-19, has emerged as a global pandemic since December 2019. While there is growing recognition regarding possible airborne transmission, particularly in the setting of aerosol-generating procedures and treatments, whether nasopharyngeal and oropharyngeal swabs for SARS-CoV-2 generate aerosols remains unclear.

Design: Systematic review.

Data Sources: We searched Ovid MEDLINE and EMBASE up to November 3 2020. We also searched the China National Knowledge Infrastructure, Chinese Medical Journal Network, medRxiv and ClinicalTrials.gov up to March 29 2020.

Eligibility Criteria: All comparative and non-comparative studies that evaluated dispersion or aerosolization of viable airborne organisms, or transmission of infection associated with nasopharyngeal or oropharyngeal swab testing.

Results: Of 7,702 citations, only one study was deemed eligible. Using a dedicated sampling room with negative pressure isolation room, personal protective equipment including N95 or higher masks, strict sterilization protocols, structured training with standardized collection methods, and a structured collection and delivery system, a tertiary care hospital proved a 0% healthcare worker infection rate among 8 nurses conducting over 11,000 nasopharyngeal swabs. No studies examining transmissibility with other safety protocols, nor any studies quantifying the risk of aerosol generation with nasopharyngeal or oropharyngeal swabs for detection of SARS-CoV-2 were identified.

Conclusions: There is limited to no published data regarding aerosol generation and risk of transmission with nasopharyngeal and oropharyngeal swabs for the detection of SARS-CoV-2.

For peer review only

Field experiments to quantify this risk are warranted. Vigilance in adhering to current standards for infection control is suggested.

For peer review only

Article summary:

Strengths and limitations of this study:

- A comprehensive literature search incorporating English and Chinese-language peer reviewed literature, grey literature and pre-print databases was conducted.
- The literature search included in-patient and outpatient testing settings.
- The literature search accounted for available direct and indirect evidence.
- Findings of this systematic review were limited to one study evaluating transmission risk to healthcare personnel conducting nasopharyngeal swabs; no studies directly quantified the risk of airborne transmission.
- The findings of the systematic review preclude substantive evidence-informed guidance, and warrant vigilance in adhering to current standards for infection prevention and control measures.

For peer review only

Background:

In late December 2019, a novel coronavirus, subsequently referred to by the World Health Organization (WHO) as *severe acute respiratory syndrome coronavirus 2* (SARS-CoV-2), was identified as the cause of atypical pneumonia cases detected in Wuhan, China (1). Since then, SARS-CoV-2 related-disease (named COVID-19), has emerged as a global pandemic (2). In one year, SARS-CoV-2 has spread to over 200 countries, infecting over 90 million individuals, and causing over 2 million deaths internationally (3).

International infection control guidance initially stated that viral transmission is likely primarily through direct, indirect or close contact with infected salivary and respiratory droplets (5 to 10 μm in diameter) (4). More recently, the WHO and the Centers for Disease Control and Prevention have recognized that there is growing evidence regarding SARS-CoV-2 transmission from airborne exposure, particularly in poorly ventilated enclosed settings with extended exposures, and in the setting of aerosol-generating procedures in healthcare settings (5-8).

Testing for COVID-19 is commonly conducted using nasopharyngeal and oropharyngeal swabs of the posterior wall of the pharynx. The risk of aerosol generation associated with obtaining nasopharyngeal or oropharyngeal swabs in patients infected with COVID-19 remains unclear. The potential to induce coughing may compound this associated risk, the extent to which is unknown.

Given thousands of nasopharyngeal and oropharyngeal swabs are conducted daily internationally, the risk of potential airborne transmission to otherwise uninfected patients and healthcare workers must be balanced with severe resource constraints in personal protective equipment. This dictates an urgent need for an updated evidence synthesis and guidance regarding the risk of aerosolization associated with swab testing. We therefore conducted a systematic review of the literature to determine the risk of aerosol generation and associated transmission associated

For peer review only

with nasopharyngeal and oropharyngeal swabs for detection of SARS-CoV-2.

For peer review only

Methods:

Prior to initiating this systematic review, WHO personnel reviewed and approved internal protocols for this systematic review. Given time constraints of the initial commissioned review for the WHO in May 2020 (7 days to completion), the protocol was not publicly registered or published.

Literature search: We conducted a comprehensive search of Ovid MEDLINE and Embase with the assistance of a health information specialist (RC) from inception to November 3 2020 (updated), using a combination of subject headings and keywords related to: respiratory tract infections including COVID-19 and other coronaviruses; swabs related to the oral and nasal cavities, nasopharynx and oropharynx; and aerosol generation and infection transmission. We did not limit the search to COVID-19 or coronavirus infection (see Appendix A for the search strategy).

We also applied the same search strategy as of March 25 2020 to the China National Knowledge Infrastructure (CNKI) and Chinese Medical Journal Network (CMJN) (YC). We did not apply any language or quality restrictions. Finally, we searched medRxiv and ClinicalTrials.gov for articles related to COVID-19 or SARS-CoV-2 for pre-prints or grey literature addressing the research question.

Selection criteria: We included all comparative and non-comparative studies that evaluated dispersion or aerosolization of viable airborne organisms or transmission of infection associated with nasopharyngeal or oropharyngeal swab testing. We included studies that evaluated outcomes including detection of viable airborne organisms through microbiological sample analysis or documented transmission of infection associated with swab testing. We initially planned to include

For peer review only

studies of hospitalized adult and pediatric patients with microbiologically confirmed COVID-19 in one or more fluid samples, of which at least one was a nasopharyngeal or oropharyngeal swab. In the absence of direct evidence meeting the eligibility criteria above, we broadened our inclusion criteria to include all study designs and populations evaluating aerosol generation or dispersion associated with swab testing. This included studies of hospitalized and non-hospitalized patients with or without microbiologically confirmed COVID-19, simulation studies without human participants, and those describing dispersion of non-infectious air particles or liquid droplets.

<u>Study selection:</u> Paired reviewers (SMF, KH, LB, SB) independently screened citations and conducted full-text review of all potentially eligible studies. Reviewers screened the reference lists of review articles to identify additional studies meeting eligibility criteria, and screened pre-prints and grey literature from medRxiv and ClinicalTrials.gov. Disagreements were resolved by a third reviewer (AA) at all stages. Paired reviewers (XX, NY, XL) screened citations identified from the CNKI and CMJN and resolved disagreements by discussion.

<u>Data extraction and quality assessment:</u> Paired reviewers planned to abstract data using a standardized form. We planned to assess risk of bias (RoB) using the modified Cochrane risk of bias tool for randomized controlled trials (9), the ROBINS-I tool for observational studies (10), and content expert assessments for experimental or simulation-based studies. We planned to assess the overall certainty of the evidence based on RoB, imprecision, indirectness, inconsistency and publication bias, informed by GRADE guidance (11, 12).

For peer review only - http://bmjopen.bmj.com/site/about/guidelines.xhtml

Data analysis: If insufficient data were available for pooled analyses, we planned to summarize results narratively.

For peer review only

Results:

Of the 7,702 citations identified in our search, 5,477 were screened for eligibility after duplicates were removed. Of these, 9 were considered potentially eligible requiring full-text review. After careful full-text review, we identified one observational study examining transmission rates associated with a safety protocol for nasopharyngeal swabs for detection of SARS-CoV-2 (13). This study implemented and evaluated a safety protocol for nasopharyngeal swab sampling in a tertiary care hospital in Wuhan, China. The protocol involved establishment of a special sampling room with negative pressure ventilation, training of nursing staff conducting swab tests, strict sterilization protocols, personal protective equipment including N95 or higher-level masks with standard droplet and contact precautions, and standardized swab collection and transportation methods. Based on preliminary evaluation among eight nurses conducting over 11,000 nasopharyngeal swabs, a 0% nursing staff infection rate was reported (13).

We did not apply the ROBINS-I tool, given the study was non-comparative. Overall certainty of evidence was very low, with very serious concerns regarding risk of bias, imprecision and publication bias.

No studies evaluating dispersion and aerosol generation associated with nasopharyngeal or oropharyngeal swabs, nor any studies comparing transmission using different safety protocols, were found despite broadening eligibility criteria (Figure 1).

For peer review only

Discussion

This systematic review on aerosol generation and associated risk of infection transmission with nasopharyngeal and oropharyngeal swabs in the detection of SARS-CoV-2 yielded limited to no evidence. Only one small study evaluating a preliminary safety protocol for nasopharyngeal swabs was found, which showed that standardized protocols and airborne precautions were associated with no infections among nursing staff conducting testing (13). The safety protocol implemented, which included a negative pressure room and N95 or higher masks, is not widely implemented in current practice; no data was provided regarding risk of transmission with nasopharyngeal swabs under other settings. Broadening of eligibility criteria to allow for all study designs, including non-comparative and experimental designs and all populations regardless of COVID-19 infection, also did not yield any relevant citations.

Airborne transmission is different from droplet transmission, as it refers to the presence of microbes within droplet nuclei. These are generally considered to be particles <5μm in diameter, which can remain in the air for periods of time, and can subsequently be transmitted to others over distances greater than 1 meter (6). Reduction of respiratory particles to <5 μm involves dehydration of larger droplets and consequent dehydration of any organisms within the droplet. If airborne particles are inhaled, they may rehydrate in the upper airway, increase in size and deposit in the airway. Thus, airborne transmission of viable organisms requires the organism to survive a process of desiccation and aerosolization in sufficient quantity to incite infection. This property is specific to the organism (14, 15).

Aerosol generating procedures may expose healthcare workers and other contacts to pathogens, and the potential for transmission of respiratory infections. The risk of aerosolization and transmission with swab testing remains unclear, particularly in the context of COVID-19. The

For peer review only - http://bmjopen.bmj.com/site/about/guidelines.xhtml

most important theoretical risk associated with testing is induction of coughing with possible droplet release and aerosolization. A previous study evaluating voluntary cough-generated aerosolization of influenza virus using a bioaerosol cyclone sampler found that 81% of participants produced detectable viral RNA, with 23% of the RNA contained in particles 1 to 4 micrometres in size and 42% contained in particles less than 1 micrometre (16).

Specific to COVID-19, experimental studies have shown aerosols with SARS-CoV-2 virus RNA in air samples for up to 3 to 16 hours (17, 18); however, studies from healthcare settings with symptomatic COVID-19 patients have yielded mixed results regarding identification of SARS-CoV-2 in air samples, and none have identified viable virus (6).

Initial guidance from the WHO on March 29, 2020 recommended droplet and contact precautions for those people caring for COVID-19 patients, and airborne precautions for settings in which aerosol generating procedures and treatments are performed (4). Subsequent guidance on December 1 2020 recommended, in addition to standard droplet and contact precautions, that medical masks in the absence of aerosol generating procedures, and respirators, N95s, or equivalent level masks be worn in care settings where such procedures are performed. Aerosol generating procedures were defined as "tracheal intubation, non-invasive ventilation, tracheotomy, cardiopulmonary resuscitation, manual ventilation before intubation, bronchoscopy, sputum induction using nebulized hypertonic saline, and dentistry and autopsy procedures" (7). Guidance specific to nasopharyngeal and oropharyngeal swab testing were not explicitly provided.

Recent evidence regarding salivary nucleic acid amplification testing has supported comparable diagnostic accuracy, with a pooled sensitivity of 83.2% (95% confidence intervals 74.7 to 91.4%) and specificity of 99.2% (98.2 to 99.8%), compared to nasopharyngeal swab testing (84.8% [76.8% to 92.4%] and 98.9% [97.4% to 99.8%]), respectively (19). While demonstrating

For peer review only

comparable yield, salivary testing was associated with lower costs (20). Coupled with increased ease of testing, reduced invasiveness and likely reduced occupational exposure risk, salivary testing may be a viable alternative to nasopharyngeal or oropharyngeal swab testing.

Taken together, recommendations regarding infection prevention and control measures during nasopharyngeal or oropharyngeal swab testing for SARS-CoV-2 should be carefully considered in light of the unknown risk of airborne transmission of infection to healthcare workers and other patients. Field experiments with nasopharyngeal and oropharyngeal swab testing in confirmed COVID-19 patients are required to adequately assess this risk using viral samplers, reverse transcriptase polymerase chain reaction testing, and viral cultures. While we cannot estimate the risk of airborne transmission, certain factors may abrogate it: adequate room ventilation, limiting exposure to the patient, high filtration fit-tested respirators (e.g. N95, FFP2) and low viral shedding (13, 21). However, the risks ought to be weighed against limited resource availability and the need for judicious use of personal protective equipment. One may also consider the yield of various fluid samples and balance their associated risks of aerosolization with this consideration. The emergence of diagnostic tests such as salivary testing with less potential for aerosol generation may diminish the enthusiasm for nasopharyngeal and oropharyngeal swabs.

Strengths of this systematic review include a comprehensive literature search incorporating English and Chinese studies, as well as grey literature and pre-prints and consultation with experts in the field. Limitations include a lack of published evidence informing the risk of aerosolization with nasopharyngeal and oropharyngeal swab testing, including indirect evidence. This highlights an important gap in the existing literature and precludes development of evidence-informed guidance beyond recommendations based on expert opinion.

For peer review only

Conclusion

Our systematic review revealed limited to no data specifically addressing the risk of aerosol generation and airborne transmission associated with nasopharyngeal and oropharyngeal swab testing in the context of COVID-19 or related infectious respiratory illnesses. Current international guidelines recommend droplet and contact precautions during testing. Given a theoretical risk of cough-related aerosolization during testing, field experiments to quantify the risk of aerosol generation and transmission associated with swab testing are warranted. In the interim, appropriate vigilance in adhering to available standards for infection prevention and control measures is suggested. Alternative testing modalities, including salivary testing, may warrant consideration in place of nasopharyngeal and oropharyngeal testing.

For peer review only

Funding: This project was supported by the World Health Organization (WHO Registration 2020/ 1010002-0) as part of a rapid review effort related to COVID-19, through the MAGIC Evidence Ecosystem Foundation (www.magicproject.org).

Acknowledgements: We would like to thank Yaolong Chen, Xuan Yu, Nan Yang and Xufei Luo for their assistance with systematic searching and screening of evidence from the China National Knowledge Infrastructure database for additional literature related to our systematic review focused on risk of aerosol generation with HFNC.

Author Contributions: Per O. Vandvik conceived the study. Arnav Agarwal and Per O. Vandvik organized the study teams and process. Arnav Agarwal and Rachel Couban designed the search strategy. Shannon M. Fernando, Kimia Honarmand, Layla Bakaa, and Sonia Brar screened studies for eligibility, and assessed study risk of bias and certainty of the body of evidence. Arnav Agarwal addressed discrepancies in screening. Arnav Agarwal wrote the first draft of the manuscript and conducted data analysis. Arnav Agarwal, Bram Rochwerg, Neill K. Adhikari, Francois Lamontagne, Srinivas Murthy, David S.C. Hui, Charles D. Gomersall, Samira Mubareka, Janet V. Diaz, Karen E.A. Burns, John Basmaji, Fiona Muttalib, David Granton, Dipayan Chaudhuri, Devin Chetan, Malini Hu, and Per O. Vandvik critically revised the manuscript. Arnav Agarwal and Per O. Vandvik are the guarantors. All co-authors were involved in final editing of the manuscript, and approved the final draft. The corresponding author attests that all listed authors meet authorship criteria.

Patient and public involvement: No patients were involved in this study, given the severe time constraints of 7 days to completion for the initial WHO-commissioned rapid review.

Data sharing statement: No additional data available.

Ethics approval statement: Not applicable.

For peer review only

Transparency: The manuscript's guarantors (POV and AA) affirm that the manuscript is an honest, accurate, and transparent account of the recommendation being reported; that no important aspects of the recommendation have been omitted; and that any discrepancies from the recommendation as planned (and, if relevant, registered) have been explained.

Competing Interests: No competing interests declared.

For peer review only

References

1. Zhu N, Zhang D, Wang W, Li X, Yang B, Song J, et al. A novel coronavirus from patients with pneumonia in China, 2019. N Engl J Med. 2020;382(8):727-33.

2. World Health Organization. Rolling updates on coronavirus disease (COVID-19). Geneva, Switzerland: World Health Organization; 2020.

3. John Hopkins Coronavirus Resource Centre. Coronavirus COVID-19 global cases by the Center for Systems Science and Engineering (CSSE) at John Hopkins University. Baltimore, Maryland: John Hopkins University; 2020.

4. World Health Organization. Modes of transmission of virus causing COVID-19: implications for IPC precaution recommendations. Geneva, Switzerland: World Health Organization; 2020.

5. Centers for Disease Control and Prevention. Scientific brief: SARS-CoV-2 and potential airborne transmission Atlanta, Georgia: U.S. Department of Health & Human Services; 2020.

6. World Health Organization. Transmission of SARS-CoV-2: implications for infection prevention precautions. Geneva, Switzerland: World Health Organization; 2020.

7. World Health Organization. Mask use in the context of COVID-19. Geneva, Switzerland: World Health Organization; 2020.

8. World Health Organization. Q&A on infection prevention and control for health care workers caring for patients with suspected or confirmed 2019-nCoV. Geneva, Switzerland: World Health Organization; 2020.

9. Higgins JP, Altman DG, Gøtzsche PC, Jüni P, Moher D, Oxman AD, et al. The Cochrane Collaboration's tool for assessing risk of bias in randomised trials. BMJ. 2011;343:d5928.

For peer review only

10. Sterne JA, Hernán MA, Reeves BC, Savović J, Berkman ND, Viswanathan M, et al. ROBINS-I: a tool for assessing risk of bias in non-randomised studies of interventions. BMJ. 2016;355:i4919.

11. Guyatt GH, Oxman AD, Vist GE, Kunz R, Falck-Ytter Y, Alonso-Coello P, et al. GRADE: an emerging consensus on rating quality of evidence and strength of recommendations. BMJ. 2008;336(7650):924-6.

12. Morgan RL, Thayer KA, Bero L, Bruce N, Falck-Ytter Y, Ghersi D, et al. GRADE: Assessing the quality of evidence in environmental and occupational health. Environ Int. 2016;92-93:611-6.

13. Qian Y, Zeng T, Wang H, Xu M, Chen J, Hu N, et al. Safety management of nasopharyngeal specimen collection from suspected cases of coronavirus disease 2019. Int J Nurs Sci. 2020;7(2):153-6.

14. Pepper IL, Gerba CP. Aeromicrobiology. In: Pepper I, Gerba C, Gentry T, editors. Environmental Microbiology 3rd ed ed. Tucson, Arizona: Elsevier Inc; 2015. p. 89-110.

15. Cox CS. Airborne bacteria and viruses. Sci Prog. 1989;73(292 Pt 4):469-99.

16. Lindsley WG, Blachere FM, Thewlis RE, Vishnu A, Davis KA, Cao G, et al. Measurements of airborne influenza virus in aerosol particles from human coughs. PLoS One. 2010;5(11):e15100.

17. van Doremalen N, Bushmaker T, Morris DH, Holbrook MG, Gamble A, Williamson BN, et al. Aerosol and surface stability of SARS-CoV-2 as compared with SARS-CoV-1. N Engl J Med. 2020;382(16):1564-7.

For peer review only

18. Fears AC, Klimstra WB, Duprex P, Hartman A, Weaver SC, Plante KS, et al. Persistence of severe acute respiratory syndrome coronavirus 2 in aerosol suspensions. Emerg Infect Dis. 2020;26(9).

19. Butler-Laporte G, Lawandi A, Schiller I, Yao MC, Dendukuri N, McDonald EG, et al. Comparison of saliva and nasopharyngeal swab nucleic acid amplification testing for detection of SARS-CoV-2: a systematic review and meta-analysis. JAMA Intern Med. 2021 [Epub ahead of print].

20. Bastos ML, Perlman-Arrow S, Menzies D, Campbell JR. The sensitivity and costs of testing for SARS-CoV-2 infection with saliva versus nasopharyngeal swabs: a systematic review and meta-analysis. Ann Intern Med. 2021 [Epub ahead of print].

21. Fennelly KP, Nardell EA. The relative efficacy of respirators and room ventilation in preventing occupational tuberculosis. Infect Control Hosp Epidemiol. 1998;19(10):754-9.

For peer review only

Figure legends

Figure 1: PRISMA flow diagram for review on aerosol generation associated with nasopharyngeal and oropharyngeal swabs.

For peer review only

Figure 1: PRISMA flow diagram for review on aerosol generation associated with nasopharyngeal and oropharyngeal swabs.

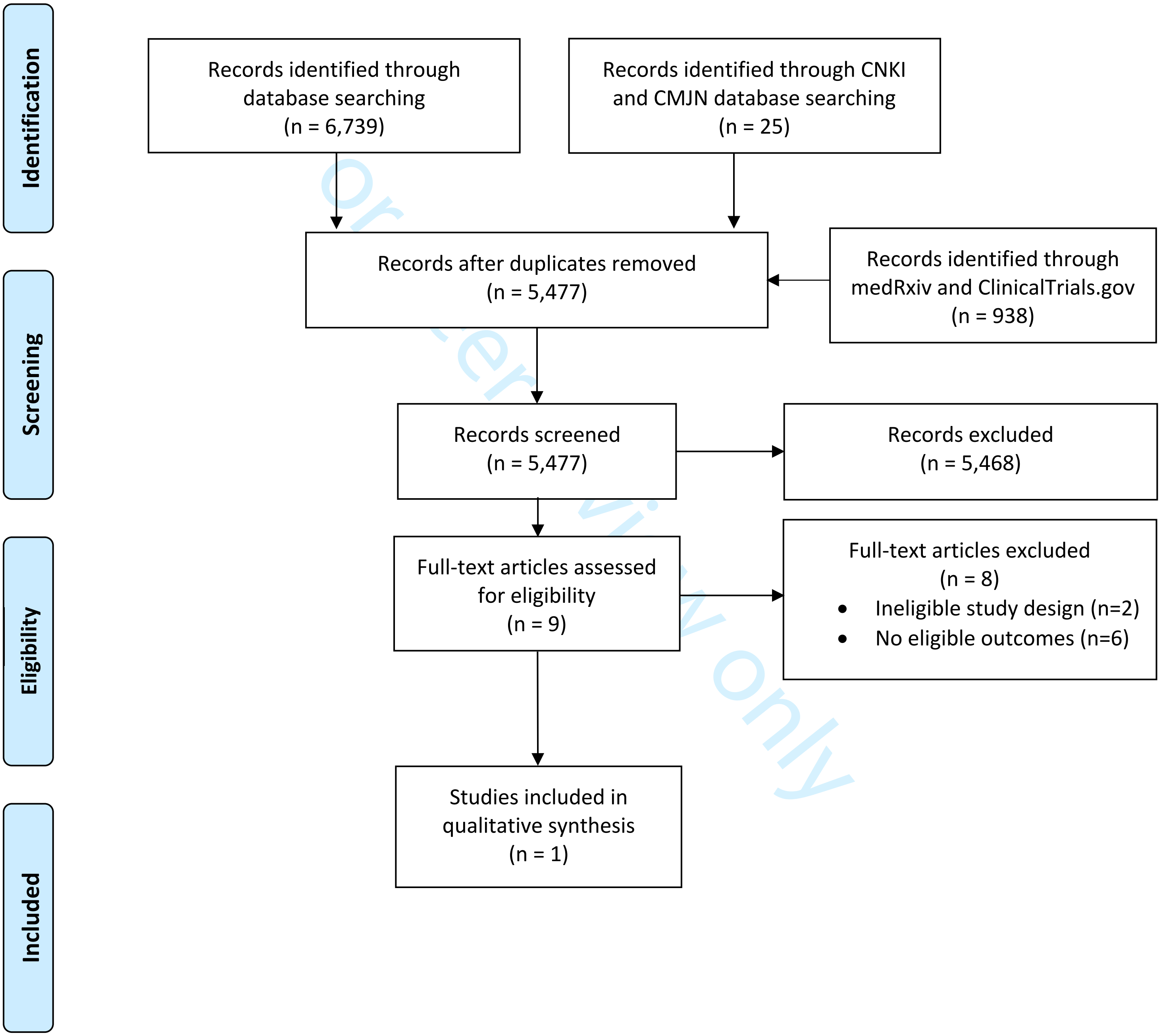

Appendix A: Search strategy for OVID Medline and EMBASE.

MEDLINE

Database: OVID Medline Epub Ahead of Print, In-Process & Other Non-Indexed Citations, Ovid MEDLINE(R) Daily and Ovid MEDLINE(R) 1946 to Present

Search Strategy:

----

1 exp Respiratory Tract Infections/ (352559)

2 (respiratory adj3 infect*).mp. [mp=title, abstract, original title, name of substance word, subject heading word, floating sub-heading word, keyword heading word, organism supplementary concept word, protocol supplementary concept word, rare disease supplementary concept word, unique identifier, synonyms] (75025)

3 (influenza* or flu).tw. (121359)

4 (h1n1 or h5n1 or h3n2).tw. (25097)

5 exp Coronavirus/ (11467)

6 exp Coronavirus Infections/ (9776)

7 (coronavir* or coronovir* or SARS or MERS or MERS-COV or SARS-COV or SARS-COV-2 or COV or NCOV or 2019nCOV or 2019-nCOV or COVID-19).mp. [mp=title, abstract, original title, name of substance word, subject heading word, floating sub-heading word, keyword heading word, organism supplementary concept word, protocol supplementary concept word, rare disease supplementary concept word, unique identifier, synonyms] (24352)

8 or/1-7 (469619)

9 ((throat or pharynx or pharyng* or oral or oropharyng* or nasal or nares or nasopharyng*) adj3 (swab* or detect* or test or sample* or specimen* or diagnos* or assay)).mp. (49581)

10 8 and 9 (8147)

11 exp Disease Transmission, Infectious/ (67129)

12 (contamina* or dispers* or droplet* or partic* or aerosol* or environment* or transmiss* or transmit or safety* or commun* or contagi*).mp. (5570978)

13 11 or 12 (5584372)

14 10 and 13 (2488)

EMBASE

Database: Embase <1974 to 2020 March 24>

Search Strategy:

----

1 exp respiratory tract infection/ (385263)

2 (respiratory adj3 infect*).mp. (127592)

3 (influenza* or flu).tw. (139944)

4 (h1n1 or h5n1 or h3n2).tw. (30836)

5 exp coronavirinae/ (11209)

6 exp Coronavirus infection/ (11152)

7 (coronavir* or coronovir* or SARS or MERS or MERS-COV or SARS-COV or SARS-COV-2 or COV or NCOV or 2019nCOV or 2019-nCOV or COVID-19).mp. (28959)

8 or/1-7 (491703)

9 ((throat or pharynx or pharyng* or oral or oropharyng* or nasal or nares or nasopharyng*) adj3 (swab* or detect* or test or sample* or specimen* or diagnos* or assay)).mp. (80034)

For peer review only

10 8 and 9 (9252)
11 disease transmission/ (96856)
12 (contamina* or dispers* or droplet* or partic* or aerosol* or environment* or transmiss* or transmit or safety* or commun* or contagi*).mp. (7223561)
13 11 or 12 (7223561)
14 10 and 13 (3189)

For peer review only

PRISMA 2009 Checklist

Section/topic	#	Checklist item	Reported on page #
**TITLE**			
Title	1	Identify the report as a systematic review, meta-analysis, or both.	1
**ABSTRACT**			
Structured summary	2	Provide a structured summary including, as applicable: background; objectives; data sources; study eligibility criteria, participants, and interventions; study appraisal and synthesis methods; results; limitations; conclusions and implications of key findings; systematic review registration number.	2-4
**INTRODUCTION**			
Rationale	3	Describe the rationale for the review in the context of what is already known.	5-6
Objectives	4	Provide an explicit statement of questions being addressed with reference to participants, interventions, comparisons, outcomes, and study design (PICOS).	5-6
**METHODS**			
Protocol and registration	5	Indicate if a review protocol exists, if and where it can be accessed (e.g., Web address), and, if available, provide registration information including registration number.	7
Eligibility criteria	6	Specify study characteristics (e.g., PICOS, length of follow-up) and report characteristics (e.g., years considered, language, publication status) used as criteria for eligibility, giving rationale.	7-8
Information sources	7	Describe all information sources (e.g., databases with dates of coverage, contact with study authors to identify additional studies) in the search and date last searched.	7
Search	8	Present full electronic search strategy for at least one database, including any limits used, such that it could be repeated.	7
Study selection	9	State the process for selecting studies (i.e., screening, eligibility, included in systematic review, and, if applicable, included in the meta-analysis).	8
Data collection process	10	Describe method of data extraction from reports (e.g., piloted forms, independently, in duplicate) and any processes for obtaining and confirming data from investigators.	8
Data items	11	List and define all variables for which data were sought (e.g., PICOS, funding sources) and any assumptions and simplifications made.	8
Risk of bias in individual studies	12	Describe methods used for assessing risk of bias of individual studies (including specification of whether this was done at the study or outcome level), and how this information is to be used in any data synthesis.	8
Summary measures	13	State the principal summary measures (e.g., risk ratio, difference in means).	9
Synthesis of results	14	Describe the methods of handling data and combining results of studies, if done, including measures of consistency (e.g., I^2) for each meta-analysis.	9

For peer review only - http://bmjopen.bmj.com/site/about/guidelines.xhtml

PRISMA 2009 Checklist

Section/topic	#	Checklist item	Reported on page #
Risk of bias across studies	15	Specify any assessment of risk of bias that may affect the cumulative evidence (e.g., publication bias, selective reporting within studies).	8
Additional analyses	16	Describe methods of additional analyses (e.g., sensitivity or subgroup analyses, meta-regression), if done, indicating which were pre-specified.	N/A
**RESULTS**			
Study selection	17	Give numbers of studies screened, assessed for eligibility, and included in the review, with reasons for exclusions at each stage, ideally with a flow diagram.	10
Study characteristics	18	For each study, present characteristics for which data were extracted (e.g., study size, PICOS, follow-up period) and provide the citations.	10
Risk of bias within studies	19	Present data on risk of bias of each study and, if available, any outcome level assessment (see item 12).	10
Results of individual studies	20	For all outcomes considered (benefits or harms), present, for each study: (a) simple summary data for each intervention group (b) effect estimates and confidence intervals, ideally with a forest plot.	10
Synthesis of results	21	Present results of each meta-analysis done, including confidence intervals and measures of consistency.	10
Risk of bias across studies	22	Present results of any assessment of risk of bias across studies (see Item 15).	10
Additional analysis	23	Give results of additional analyses, if done (e.g., sensitivity or subgroup analyses, meta-regression [see Item 16]).	10
**DISCUSSION**			
Summary of evidence	24	Summarize the main findings including the strength of evidence for each main outcome; consider their relevance to key groups (e.g., healthcare providers, users, and policy makers).	11
Limitations	25	Discuss limitations at study and outcome level (e.g., risk of bias), and at review-level (e.g., incomplete retrieval of identified research, reporting bias).	13
Conclusions	26	Provide a general interpretation of the results in the context of other evidence, and implications for future research.	14
**FUNDING**			
Funding	27	Describe sources of funding for the systematic review and other support (e.g., supply of data); role of funders for the systematic review.	15

From: Moher D, Liberati A, Tetzlaff J, Altman DG, The PRISMA Group (2009). Preferred Reporting Items for Systematic Reviews and Meta-Analyses: The PRISMA Statement. PLoS Med 6(7): e1000097. doi:10.1371/journal.pmed1000097

For more information, visit: www.prisma-statement.org.

For peer review only - http://bmjopen.bmj.com/site/about/guidelines.xhtml
